# Development of a nomogram for identifying periodontitis cases in Denmark

**DOI:** 10.1038/s41598-024-60624-3

**Published:** 2024-05-17

**Authors:** Luisa Schertel Cassiano, Anne B. Jensen, Julie B. Pajaniaye, Fábio R. M. Leite, Huihua Li, Anette Andersen, Fernando V. Bitencourt, Gustavo G. Nascimento

**Affiliations:** 1 Section for Oral Ecology, Department of Dentistry and Oral Health, Aarhus University, Vennelyst Boulevard 9, 8000 Aarhus, Denmark; 2 National Dental Research Institute Singapore, National Dental Centre Singapore, Singapore, Singapore; 3 Oral Health Academic Clinical Programme, Duke-NUS Medical School, Singapore, Singapore; 4 Steno Diabetes Center Aarhus, Aarhus University Hospital, Aarhus, Denmark

**Keywords:** Oral diseases, Periodontitis

## Abstract

Although self-reported health outcomes are of importance, attempts to validate a clinical applicable instrument (e.g., nomogram) combining sociodemographic and self-reported information on periodontitis have yet to be performed to identify periodontitis cases. Clinical and self-reported periodontitis, along with sociodemographic data, were collected from 197 adults. Akaike information criterion models were developed to identify periodontitis, and nomograms developed based on its regression coefficients. The discriminatory capability was evaluated by receiver-operating characteristic curves. Decision curve analysis was performed. Smoking [OR 3.69 (95%CI 1.89, 7.21)], poor/fair self-rated oral health [OR 6.62 (95%CI 3.23, 13.56)], previous periodontal treatment [OR 9.47 (95%CI 4.02, 22.25)], and tooth loss [OR 4.96 (95%CI 2.47, 9.97)], determined higher probability of having "Moderate/Severe Periodontitis". Age [OR 1.08 (95%CI 1.05, 1.12)], low educational level [OR 1.65 (95%CI 1.34, 2.23)], poor/fair self-rated oral health [OR 3.57 (95%CI 1.82, 6.99)], and previous periodontal treatment [OR 6.66 (95%CI 2.83, 15.68)] determined higher probability for "Any Periodontitis". Both nomograms showed excellent discriminatory capability (AUC of 0.83 (95%CI 0.75, 0.91) and 0.81 (95% CI 0.74, 0.88), good calibration, and slight overestimation of high risk and underestimation of low risk. Hence, our nomograms could help identify periodontitis among adults in Denmark.

## Introduction

Patient-reported outcomes (PROs) and patient-reported outcome measures (PROMs), such as self-reported health, provide valuable information about an individual’s overall perception of health and support a person-centered approach^1,2^. While PROs and PROMs have been used in several medical fields, dentistry has lagged far behind, and most attempts to use self-perceived oral health have been made to replace clinical diagnoses^3^. However, the validation of self-reported oral conditions has yielded conflicting results, mainly because self-perception is highly dependent on past disease experience^4^ and sociodemographic factors, including age, income, and education^5,6^.

While the value of the clinical examination remains undisputable, from a public health perspective, self-reporting could be a valuable tool for assessing the prevalence of oral conditions and managing their impact on a population level. In a recent publication, we proposed the use of PROs and PROMs for screening purposes as an attempt to maximize resource allocation, decrease the paradigm of under-/overdiagnosis and under-/overtreatment, and reduce oral health inequalities^3^. Even though PROs have been receiving more attention lately, their practical integration beyond research scenarios has seen limited progress^7,8^.

Periodontitis, a progressive inflammatory disease that affects the supporting tissues of the teeth, continues to pose a significant public health challenge. Despite the decline in dental caries, the prevalence of periodontitis remained steady from 1990 to 2019, affecting approximately 45% of the global adult population^9^. Beyond the substantial costs imposed on society in the order of 154 billion dollars in the United States and over 158 billion euros in Europe^10^, periodontitis reduces the quality of life by undermining essential physical and psychosocial functions.

Efforts to validate self-reported periodontitis have considered multiple factors, such as the choice of the periodontal classification and the disease severity. For instance, the Centers for Disease Control and Prevention and the American Academy of Periodontology (CDC/AAP) proposed an 8-item instrument to assess periodontitis in the American population as part of the National Health and Nutrition Examination Survey (NHANES) cycle 2009–2010^11^. The proposed instrument showed adequate performance in assessing the prevalence of periodontitis using the CDC/AAP periodontal criteria. Noteworthy, the accuracy of the model improved upon the inclusion of socioeconomic and behavioral information, even when utilizing a reduced number of the original 8-item self-reported periodontal questionnaire^11^. On a similar note, Leite and colleagues demonstrated that the addition of socioeconomic and systemic health information was crucial in predicting periodontal disease cases^12^.

Nomograms, derived from multivariable logistic regression modeling, serve as valuable instruments for displaying the predicted probabilities of specific events facilitating communication between healthcare professionals and individuals^13^. These tools are widely utilized in various medical fields, particularly for cancer prognosis^14^. Nevertheless, the use of nomograms in oral health is meager, with this study likely representing one of the initial attempts to assess their performance using a self-reported periodontitis questionnaire in a sample characterized by a broader age range and diverse socioeconomic backgrounds. A previous study by Sim and coworkers constructed a nomogram to screen severe cases of periodontitis among individuals referred to a tertiary specialty center in Singapore, undermining the generalizability of their findings^15^.

Accordingly, this study aimed to construct a nomogram for identifying periodontitis cases among adults living in Denmark using the 8-item instrument proposed by the CDC/AAP alongside sociodemographic parameters versus the clinical periodontal diagnosis.

## Results

**Figure 1:**
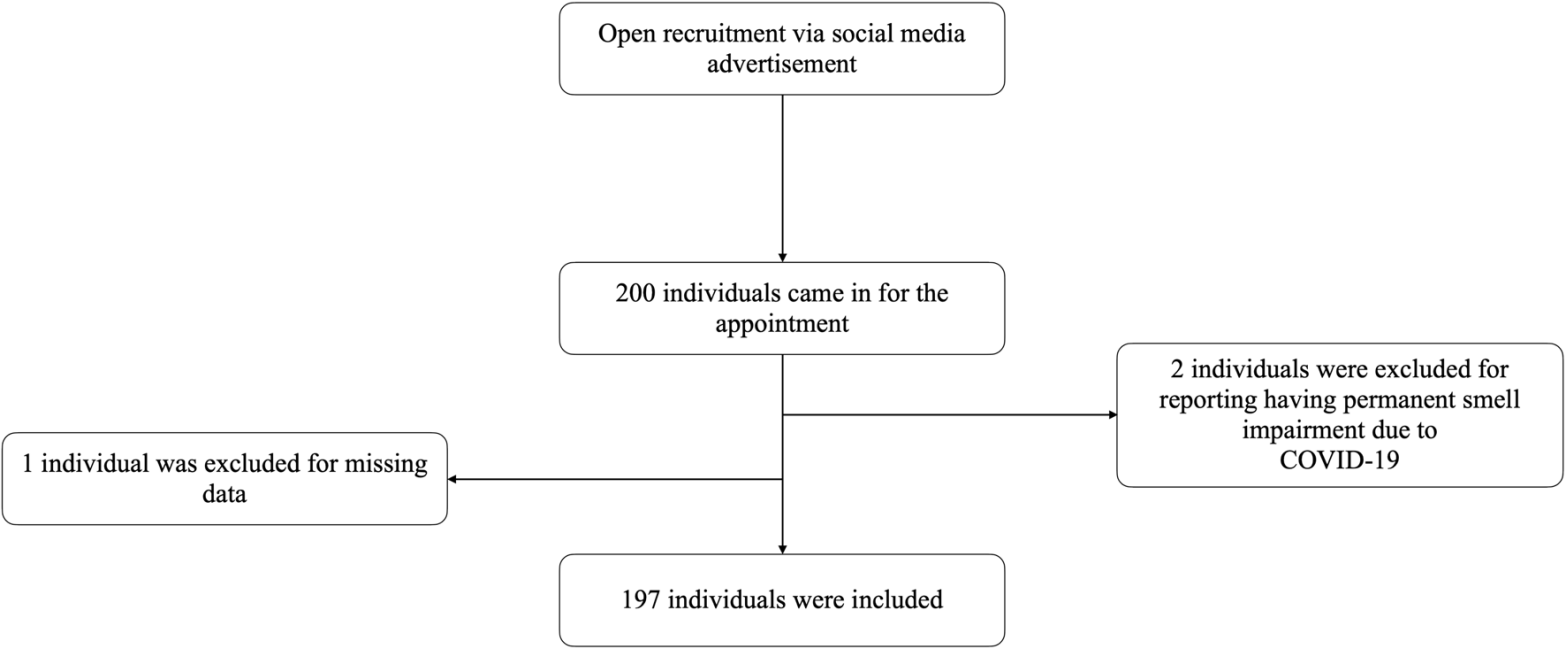
Flowchart of inclusion/exclusion criteria and the final number of patients included in the study.

A descriptive analysis of our sample is presented based on the classification of periodontitis, in Tables 1 (“Moderate/severe Periodontitis”) and Table 2 (“Any Periodontitis”). A sample of 200 individuals was recruited, however, two participants did not meet the inclusion criteria and were excluded in addition to 1 participant who did not have complete data for the variables used in the study (Fig. 1). Approximately 25% of our sample presented “Moderate/severe periodontitis”, and among those, 66% were females, and the mean age was 49.2 years (Table 1). When considering “Any periodontitis”, 34% of the sample reported having periodontitis, and 69% were females, and mean age of 49.9 years (Table 2).

**Table 1:** Prediction of “Moderate/severe Periodontitis” by logistic regression.

	No of Patients without periodontal disease	N^o^ of Patients with periodontal disease	Univariable regression		Multivariable regression	
			OR (95% CI)	*P* value	OR (95% CI)	*P* value
Age	147	50	1.06 (1.03, 1.09)	**0.001**		
Sex						
Female	109	33	Reference			
Male	38	17	1.48 (0.74, 2.95)	0.269		
Education level						
Up to high school	36	19	Reference			
University degree	111	31	0.53 (0.27, 1.05)	0.068		
Smoking						
No	107	21	Reference		Reference	
Yes	40	29	3.69 (1.89, 7.21)	** < 0.001**	2.88 (1.23, 6.73)	**0.015**
Diabetes family history						
No	98	34	Reference			
Yes	49	16	0.94 (0.47, 1.87)	0.862		
Hipertension						
No	143	46	Reference			
Yes	4	4	3.11 (0.75, 12.93)	0.059		
Gum disease						
No	134	28	Reference			
Yes	3	15	23.93 (6.49, 88.22)	** < 0.001**		
Oral health						
Good/Very good/Excellent	124	23	Reference		Reference	
Poor/Fair	22	27	6.62 (3.23, 13.56)	** < 0.001**	5.07 (2.08, 12.35)	** < 0.001**
Bone loss						
No	137	41	Reference			
Yes	4	4	3.34 (0.8, 13.95)	0.098		
Previous treatment						
No	125	24	Reference		Reference	
Yes	11	20	9.47 (4.02, 22.28)	** < 0.001**	5.83 (2.17, 15.64)	** < 0.001**
Tooth loss						
No	100	15	Reference		Reference	
Yes	47	35	4.96 (2.47, 9.97)	** < 0.001**	3.29 (1.39, 7.76)	**0.007**
Treatment in past 3 months						
No	135	41	Reference			
Yes	7	5	2.35 (0.71, 7.81)	0.162		
Flossing						
No	34	7	Reference			
Yes	113	43	1.85 (0.76, 4.48)	0.174		
Mouthwash						
No	133	42	Reference			
Yes	14	8	1.81 (0.71, 4.61)	0.214		
Bad breath						
Never/Rarely	70	19	Reference			
Sometimes/Often	77	31	1.48 (0.77, 2.86)	0.239		

Significant values are in [bold].

**Table 2:** Prediction of “Any Periodontitis” by logistic regression.

	N^o^ of Patients without periodontal disease	N^o^ of Patients with periodontal disease	Univariable regression		Multivariable regression	
			OR (95% CI)	*P* value	OR (95% CI)	*P* value
Age	129	68	1.08 (1.05, 1.12)	** < 0.001**	1.09 (1.05, 1.13)	** < 0.001**
Sex						
Female	95	47	Reference			
Male	34	21	1.25 (0.65, 2.38)	0.501		
Education level						
Up to high school	32	23	Reference		Reference	
University degree	97	45	0.65 (0.34, 1.23)	0.181	0.32 (0.13, 0.80)	**0.015**
Smoking						
No	92	36	Reference			
Yes	37	32	2.21 (1.2, 4.07)	**0.011**		
Diabetes family history						
No	84	48	Reference			
Yes	45	20	0.78 (0.41, 1.47)	0.438		
Hipertension						
No	126	63	Reference			
Yes	3	5	3.41 (0.80, 14.74)	0.0500		
Gum disease						
No	117	45	Reference			
Yes	2	16	20.8 (4.6, 94.12)	** < 0.001**		
Oral health						
Good/Very good/Excellent	107	40	Reference		Reference	
Poor/Fair	21	28	3.57 (1.82, 6.99)	** < 0.001**	3.96 (1.60, 9.81)	**0.003**
Bone loss						
No	120	58	Reference			
Yes	4	4	2.07 (0.5, 8.57)	0.316		
Previous treatment						
No	109	40	Reference		Reference	
Yes	9	22	6.66 (2.83, 15.68)	** < 0.001**	4.43 (1.66, 11.81)	**0.003**
Tooth loss						
No	91	24	Reference			
Yes	38	44	4.39 (2.35, 8.2)	** < 0.001**		
Treatment in past 3 months						
No	117	59	Reference			
Yes	7	5	1.42 (0.43, 4.65)	0.566		
Flossing						
No	33	8	Reference			
Yes	96	60	2.58 (1.12, 5.95)	**0.027**		
Mouthwash						
No	115	60	Reference			
Yes	14	8	1.10 (0.44, 2.76)	0.847		
Bad breath						
Never/Rarely	62	27	Reference			
Sometimes/Often	67	41	1.41 (0.77, 2.55)	0.263		

Significant values are in [bold].

### Moderate/severe periodontitis

**Figure 2:**
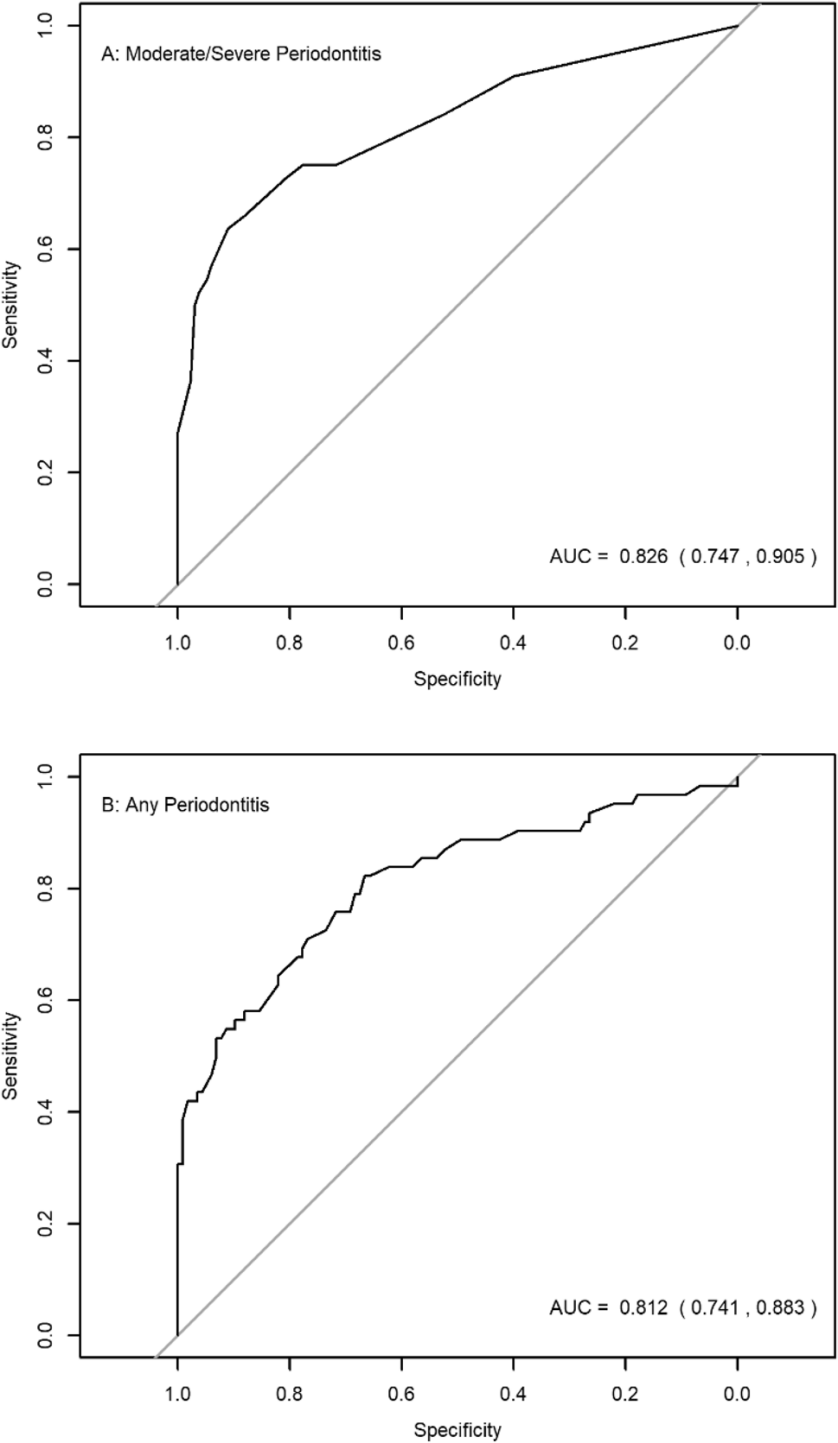
Receiver Operating Characteristic curve in A predicting “Moderate/Severe Periodontitis” and B predicting “Any Periodontitis”.

Questions on age, smoking, history of gum disease, self-rated oral health, previous periodontal treatment, and tooth loss showed a significant effect on the discrimination of “Moderate/Severe Periodontitis” by univariable logistic regression (Table 1). Based on multivariable logistic regression, participants who smoked or were former smokers, reported poor/fair oral health, with a history of periodontal treatment and exhibited tooth loss had a higher probability of having “Moderate/Severe Periodontitis” (Table 1). The ROC curve analysis showed excellent discrimination in differentiating “Moderate/severe Periodontitis” from the rest with an AUC of 0.83 [95% CI = (0.75, 0.91)] (Fig. 2A). The optimal cut-off risk of predicted probabilities was 0.26, providing a sensitivity of 72.7% and specificity of 80.7%.

**Figure 3:**
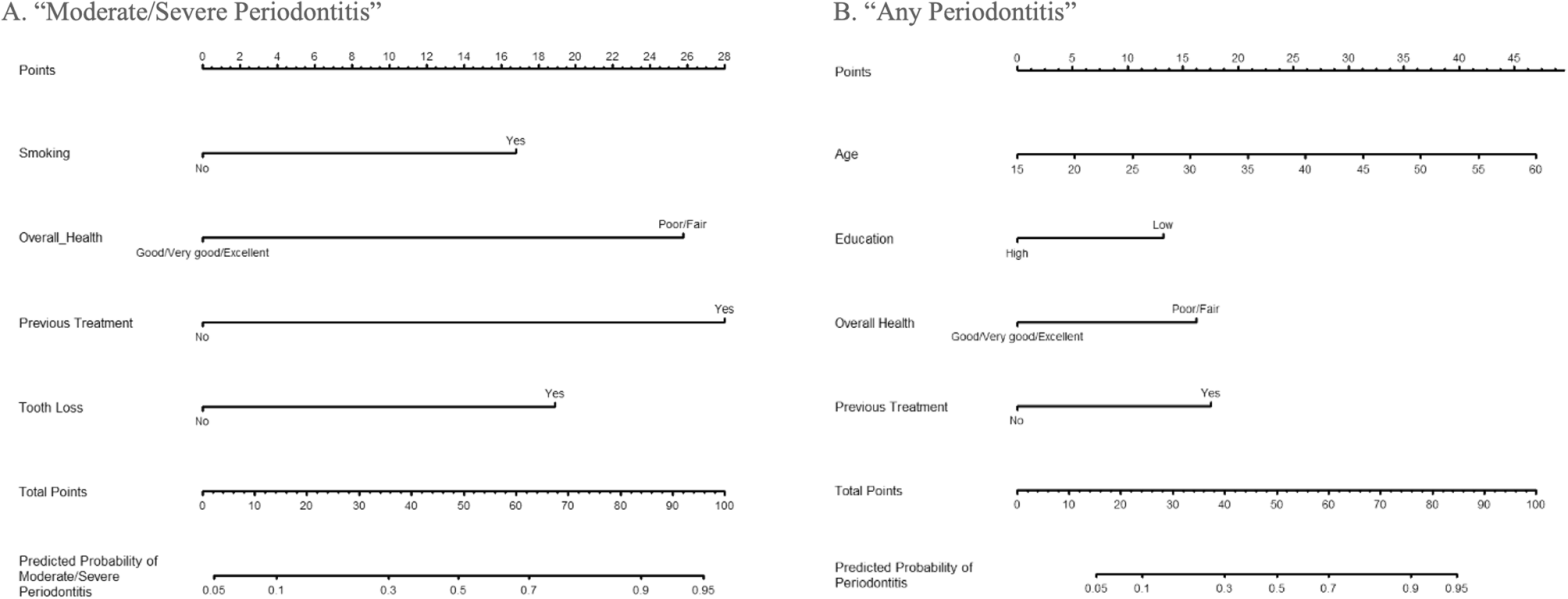
Nomogram A predicting “Moderate/Severe Periodontitis” and B predicting “Any Periodontitis”. To use the nomogram, an individual participant’s value is located on each variable axis, and a line is drawn upward to determine the number of points received for each variable value. The sum of these numbers is located on the Total Points axis to determine the risk of moderate/severe periodontitis (**A**) or any periodontitis (**B**).

A nomogram to predict “Moderate/Severe Periodontitis” was developed based on this model (Fig. 3A). The upper part of the nomogram (‘Points’) is used to compute the weight of every factor in this nomogram (smoking, self-rated oral health, previous periodontal treatment, and tooth loss). For example, the nomogram can predict the probability of having “Moderate/Severe Periodontitis” for a non-smoker (0 points) with poor self-rated oral health (26 points) and tooth loss (19 points) but no previous history of periodontal treatment (0 points). The sum of these scores (26 + 19 + 0 + 0) gives a ‘Total Score’ of 45, which is equivalent to a probability of 43.2% of having “Moderate/Severe Periodontitis” given the cut-off of 26%. In this case, this individual will be considered to have a high probability of having “Moderate/Severe Periodontitis”, suggesting the need for a referral for further clinical assessment.

**Figure 4:**
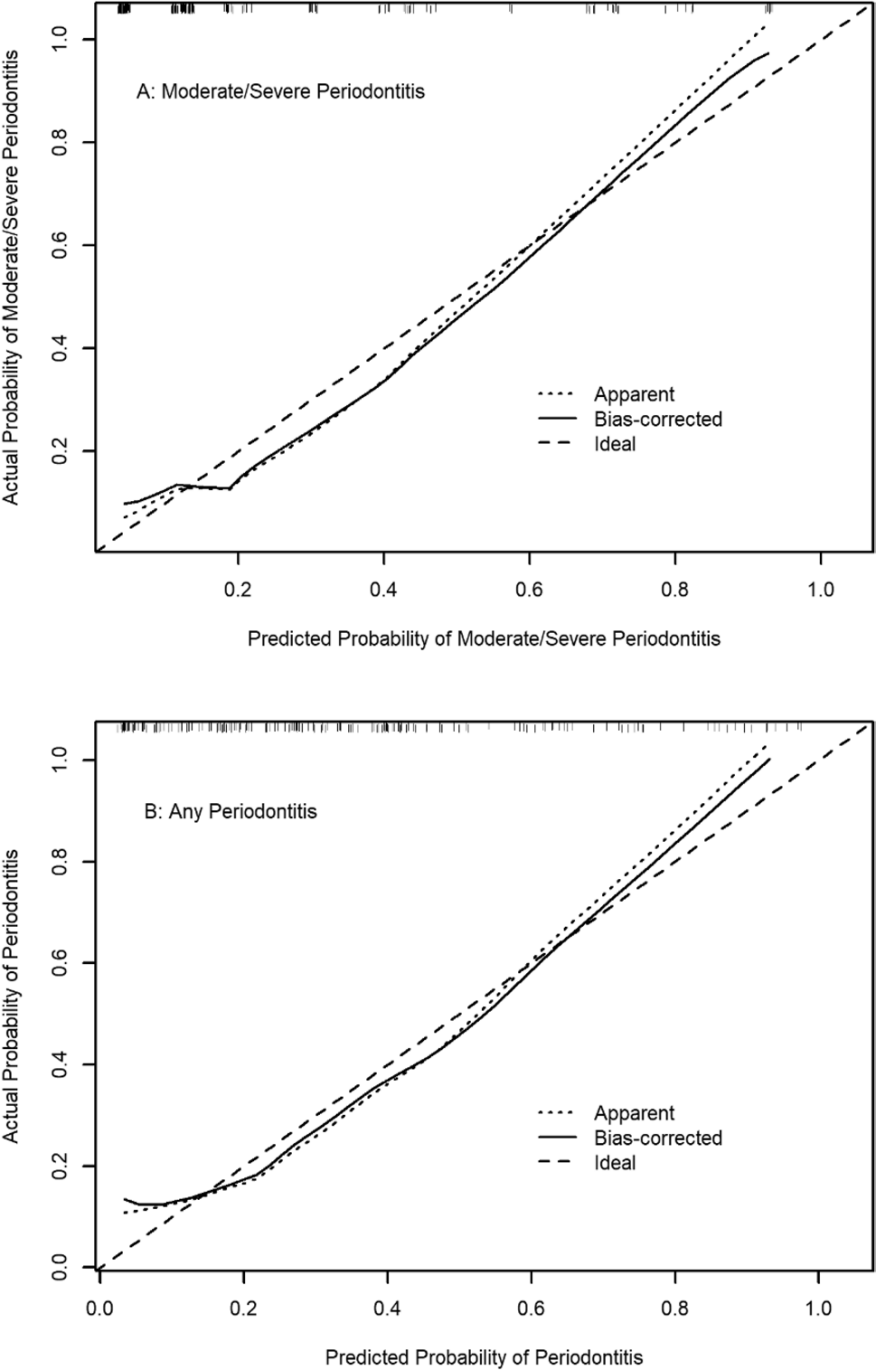
Calibration curves for AIC Selected Models, A: “Moderate/Severe Periodontitis”, B: “Any Periodontitis”. The diagonal dotted line represents a perfect prediction by an ideal model. The solid line represents the performance of the nomogram, for which a closer fit to the diagonal dotted line represents a better prediction.

**Figure 5:**
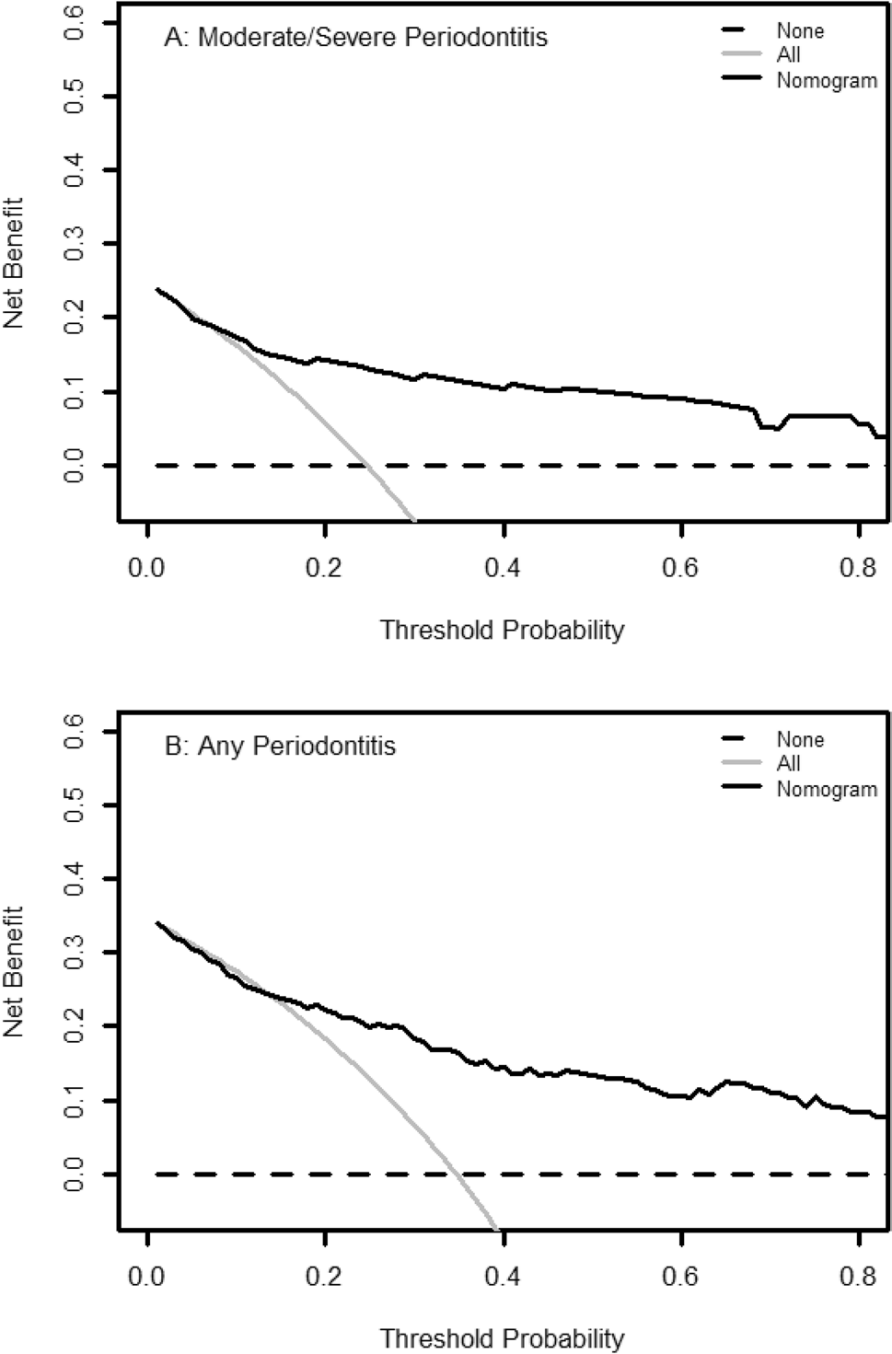
The decision curve plotting of net benefit against threshold probability for nomogram (**A**) predicting “Moderate/Severe Periodontitis”, (**B**) predicting “Any Periodontitis”.

Logistic regression analysis showed that the probability of having “Moderate/Severe Periodontitis” increased with an increasing nomogram score [OR = 1.06, 95% CI = (1.05, 1.09)]. The Hosmer–Lemeshow goodness-of-fit tests showed a good fit between observed and predicted events using this nomogram (*p* = 0.635). The calibration curve demonstrated good bootstrap estimates of calibration accuracy with an intercept close to 0 (-0.062) and a slight overestimation of high risk and underestimation of low risk (slope = 0.932) (Fig. 4A). These findings were confirmed by internal validation using bootstrapping (average bootstrap AUC = 0.81). DCA consistently showed a favorable clinical net benefit across a broad range of threshold probabilities when compared to the assumption of 'no participant' or 'all participants' with “Moderate/Severe Periodontitis” (Fig. 5A).

### Any periodontitis

Multivariable logistic regression revealed that older participants, those who reported poor/fair oral health, and those with a history of previous periodontal treatment tended to have a higher probability of having periodontitis, whereas those with a low education tended to have a higher probability of having periodontitis (Table 2). The ROC curve analysis showed similar discrimination in differentiating “Any Periodontitis” from the rest with an AUC of 0.81 [95% CI = (0.74, 0.88)] (Fig. 2B). The optimal cut-off risk of predicted probabilities (0.38) yielded a sensitivity of 71.0% and specificity of 76.9%. The resulting nomogram for predicting “Any Periodontitis” is presented in Fig. 3B.

Similarly, the probability of having periodontitis increased with an increasing nomogram score [OR = 1.09, 95% CI = (1.06, 1.12)]. The Hosmer–Lemeshow goodness-of-fit tests showed a good fit between observed and predicted events using this nomogram (*p* = 0.459). The calibration curve demonstrated good calibration accuracy with an intercept near − 0.028 and a slope of 0.928 (Fig. 4B), indicating slight overestimation of high risk and underestimation of low risk. Interval validation using bootstrapping confirmed these findings, with an average bootstrap AUC = 0.80. DCA showed consistent clinical net benefit across a wide range of threshold probabilities as well (Fig. 5B).

## Discussion

This study aimed to develop a nomogram for identifying periodontitis cases among adults living in Denmark combining the 8-item CDC/AAP questionnaire with sociodemographic information. Our results indicated a high performance of our nomogram models in identifying individuals at higher risk of having periodontitis, based on previous history of disease, as well as sociodemographic information.

We examined the CDC/AAP 8-item self-reported periodontal questionnaire^11^, alongside five additional sociodemographic questions and the clinical examination of tooth loss. Our findings highlight the significance of factors like age, history of smoking, self-rated oral health, previous periodontal treatment, and tooth loss that were significantly associated with “Moderate/Severe Periodontitis”. These same factors also hold relevance when considering “Any Periodontitis”. Our results align with a previously published study^15^, affirming the suitability of the 8-item questionnaire for identifying individuals with periodontitis.

One of the limitations of our study refers to the use of clinically assessed tooth loss in the analysis. The purpose of the nomogram is to serve as an instrument to help identify individuals with periodontitis based on self-reported information. However, it has been reported that the self‑reported number of teeth tends to agree with clinical tooth counts^6,16–19^. Therefore, we believe that using the clinical data should not have affected our results and the applicability of the nomogram. Another possible limitation of our study is that the CDC/AAP questionnaire has not been validated in Danish. However, previous studies have used it among Danish individuals^20,21^. Furthermore, our examination among our sample shows that some of the questions are significantly correlated with the presence of periodontitis. Additionally, while our nomogram has been developed on the basis of a sample originating from the general population, we did not apply methods for sample selection that would ensure its representativeness of the Danish population.

Our study’s main strengths rely on the novelty of the proposed nomograms, which, to the best of our knowledge, are the first published concerning individuals living in Denmark. In addition, our results support the use of this tool in identifying individuals with periodontitis. While most studies concerning self-reported periodontitis involve the validation of questionnaires^22,23^, our study introduces a tool with clinical utility and applicability. Notably, such a tool has only been previously published in the context of an Asian population of older persons^15^.

The nomogram for “Moderate/Severe Periodontitis” included two questions from the 8-item self-reported periodontal questionnaire (“overall oral health” and “previous periodontal treatment”), the history of smoking, and tooth loss. The other questions did not have the necessary load to be included in the nomogram. The final proposed instrument showed good discriminative capability to detect “Moderate/Severe Periodontitis” with a sensitivity of 72.7% and specificity of 80.7%, and a cut-off risk of predicted probabilities of 0.26. Information on previous periodontal treatment presented the biggest effect on identifying periodontitis among the included questions, with an OR of 9.47. These results seem to agree with findings from a study on a similar population, where a confirmatory analysis was performed using the data from the 8-item questionnaire, and the history of previous periodontal treatment was one of the questions that identified periodontitis^20^. The nomogram for “Any Periodontitis” included the same two questions from the 8-item questionnaire (“overall oral health” and “previous periodontal treatment”) along with age and education. This model also presented a good discriminative capability with a sensitivity of 71.0% and specificity of 76.9%, with a cut-off risk of predicted probabilities of 0.38. Furthermore, our nomograms demonstrated excellent discriminatory ability, with an AUC of 0.83 for “Moderate/Severe Periodontitis” and 0.81 for “Any Periodontitis”. These values are consistent with one previously reported^20^ and affirm their suitability for periodontitis screening.

The differences in the questions included in each of the nomograms may be attributed to variations in the severity of disease. According to our nomogram, “Any Periodontitis” could be predicted by age, education, self-rated oral health, and history of previous periodontal treatment, all well-established risk factors for periodontitis^24^. Several studies have consistently shown the effect of education, as a measure of socioeconomic position, on health outcomes, including oral health. In a study conducted among Brazilian adults, Schuch and collaborators identified that low socioeoconomic position was associated with a higher risk of periodontitis, finding consistently demonstrated in a systematic review^25,26^. As “Any Periodontitis” comprises cases from mild to severe, the identification of education as a potential predictor is not surprising. Tooth loss and smoking, however, were strong predictors of “Moderate/Severe Periodontitis” cases. The effect of smoking on periodontitis onset and progression has been documented in several observational and clinical studies^27^. It has been suggested that smoking locally and systemically affects tissues and host cells, mostly impacting the inflammatory response and healing capacity^28^. Given that smoking has a cumulative effect, its association with “Moderate/Severe Periodontitis” cases only may reflect this dose–response effect. On a similar note, tooth loss is a direct consequence of severe periodontitis, and its ability to predict severe cases of periodontal destruction is aligned with the available evidence^29^, including the AAP/EFP periodontal classification system, which considers tooth loss a criterion for periodontal diagnosis^30^. The variables identified in our nomogram appear to be aligned with a previous study, which also identified that variables such as education and tooth loss are strong indicators of periodontal destruction^12^. One concern that could arise from our data is the fact that tooth loss or previous treatment could be considered a direct consequence of periodontitis, which could defeat the nomogram, in case it should be used for disease prediction. However, the nomogram is proposed to as a tool to identify individual with periodontitis, and not a predictive tool.

Interestingly, the questions identified in our nomogram for adults living in Denmark differ from those included in an Asian study. In Singapore, the periodontal questions identified by the authors included self-reported information about bone loss, loose teeth, and the use of mouth rinse, combined with age and education to identify severe periodontitis cases. Notably, the study conducted by Sim and colleagues used a different periodontal classification system, potentially explaining the different variables identified^15^. Leite and coworkers highlighted the impact of classification systems on accuracy in predicting periodontitis occurrence. These variations between nomograms underscore the importance of evaluating the self-report measures within specific populations, accounting for population-specific characteristics, access to dental care, cross-cultural adaptation of questionnaire items, and periodontitis prevalence^12^.

Our findings showed the feasibility of using a reduced version of the CDC/AAP 8-item questionnaire alongside sociodemographic information in the form of a nomogram for screening individuals with a high probability of having periodontitis. This approach offers potential advantages, as reduced questionnaires tend to increase compliance. Additionally, the nomogram can serve as a valuable tool for collecting self-reported information on periodontitis for public health, surveillance, research purposes, population mass screening, and can potentially serve as a tool for a life-long periodontal control.

### Future work and clinical implications

The future work is focused on the use of the nomograms in research, clinical practice, epidemiological studies, surveillance, and health planning purposes, as well as in eHealth initiatives.

## Methods

Data for this study were derived from a cross-sectional observational study conducted from September 2022 to April 2023 at Aarhus University, Denmark. A sample of 198 participants was recruited through social media advertisements. Eligible participants were adults aged 18–55 years, who had a minimum of 15 teeth, and were able to attend one information meeting and a clinical visit. Individuals who agreed to participate and provided written informed consent were scheduled for data collection.

On the clinical visit, all participants completed a questionnaire about self-reported periodontitis and sociodemographic information, followed by clinical data collection on periodontitis and tooth loss. All self-reported information was collected through a questionnaire and managed using REDCap (Research Electronic Data Capture) electronic data capture tools hosted at Aarhus University^31,32^. The study was approved by the Central Denmark Region Ethics Committee (IRB #1-10-72-157-20). Additionally, all research was performed in accordance with relevant regulations following the Declaration of Helsinki.

### Periodontitis: clinical examination

The clinical parameters used to assess periodontitis were probing pocket depth (PPD), clinical attachment level (CAL), and furcation involvement (FUR). A standardized University of North Carolina 15 periodontal probe (Hu-Friedy, Chicago, IL, USA) was used for the clinical examination. Furcation defects were assessed using a Nabers no. 2 furcation probe. Recordings were collected from all present teeth except third molars, in six sites per tooth (mesiobuccal, mid-buccal, distobuccal, mesiolingual, mid-lingual, and distolingual), except for FUR that was only recorded on molars and premolars^33^.

Periodontitis was classified following the CDC/AAP classification^34^, which has demonstrated high accuracy for studies in an adult population^35^. The CDC/AAP classification groups individuals into four categories based on their periodontal scores^36^: (1) healthy subjects (that do not fit into mild, moderate, or severe categories), (2) mild periodontitis (≥ 2 interproximal sites with ≥ 3 mm clinical attachment loss and ≥ 2 interproximal sites with ≥ 4 mm periodontal probing depth (not on the same tooth) or 1 site with ≥ 5 mm periodontal probing depth), (3) moderate periodontitis (≥ 2 interproximal sites with ≥ 4 mm clinical attachment loss (not on the same tooth) or ≥ 2 interproximal sites with periodontal probing depth ≥ 5 mm (not on the same tooth), and (4) severe periodontitis (≥ 2 interproximal sites with ≥ 6 mm clinical attachment loss (not on the same tooth) and ≥ 1 or more interproximal site(s) with ≥ 5 mm periodontal probing depth). Based on this classification, we dichotomized periodontitis into two different ways: first, as “Moderate/Severe Periodontitis,” grouping individuals from categories (1) and (2) versus (3) and (4); and second, as “Any Periodontitis,” grouping individuals from categories (2), (3), and (4), while category (1) remained as the reference.

### Periodontitis: self-reported

Self-reported periodontitis was assessed using the CDC/AAP questionnaire^37^ through the following questions:

1. Do you think you might have gum disease? (Yes, No, Refused, Do Not Know)
2. Overall, how would you rate the health of your teeth and gums? (Excellent, Very good, Good, Fair, Poor, I Do Not Know)
3. Have you ever had gum disease treatments such as scaling and root planing, sometimes called “deep cleaning”? (Yes, No, Refused, Do Not Know)
4. Have you ever had any teeth become loose on their own without an injury? (Yes, No, Refused, Do Not Know)
5. Have you ever been told by a dental professional that you lost bone around your teeth? (Yes, No, Refused, Do Not Know)
6. During the past 3 months, have you noticed a tooth that does not look right? (Yes, No, Refused, Do Not Know)
7. Aside from brushing your teeth with a toothbrush, in the last 7 days, how many times did you use dental floss or any other device to clean between your teeth? (Number of days, Refused)
8. Aside from brushing your teeth with a toothbrush, in the last 7 days, how many times did you use mouthwash or other dental rinse products that you use to treat dental disease or dental problems? (Number of days, Refused)

For analytical purposes, the “Refused” and “Do Not Know” categories were treated as missing data.

### Sociodemographic and health parameters

The following self-reported information was collected: age, sex (male/female), level of education [“What is your highest level of education?” We considered low if elementary school (e.g., primary school), secondary education (e.g., high school), vocational training; and high if short higher education (up to 2 years), medium higher education (2–4.5 years) (e.g., Bachelor and Diploma programs), higher education (5 years or more) (e.g., master’s and Ph.D. programs)], smoking (“Do you smoke?” Options: I have never smoked, I am a former smoker, I am a smoker), bad breath (“Do you think you have bad breath?” Options: Never, Rarely, Sometimes, Often), and family history of diabetes [“Does anyone in your family have high blood sugar (diabetes)? Options: Yes, No].

### Statistical analysis

Univariable logistic regression was carried out to evaluate the effect of self-reported questions and socioeconomic variables on periodontitis discrimination. Multivariable models were developed considering as potential factors those with *p* value < 0.2 in the univariable regression^38^. The most suitable model was selected based on Akaike information criterion (AIC). The performance of this model was investigated to evaluate its discriminatory capability to diagnose periodontitis using a Receiver Operating Characteristic (ROC) curve. The Area Under the Curve (AUC) was reported for each model with AUC values of 0.5 indicating no discrimination, 0.7–0.8 as acceptable, 0.8–0.9 as excellent, and > 0.9 as outstanding in identifying people with the disease from the rest^39^.

Nomograms were generated based on the coefficients of the AIC-selected model. Calibration plots were generated to evaluate the ability of the nomogram to predict periodontitis of individual participants by calculating an optimism-corrected estimate of performance with 2000 bootstrap resamples. Perfect calibration is achieved when the estimated regression slope equals 1 and the intercept equals 0. A slope > 1 denotes underestimation of high risk and overestimation of low risk, while a slope < 1 denotes overestimation of high risk and underestimation of low risk. An intercept > 0 indicates an average underestimation, while an intercept < 0 indicates an average overestimation^40^. The upper part of a nomogram (‘Points’) is used to compute the weight of every factor in that nomogram. Then the sum of these points, reflected on the ‘Total Points’ axis located at the lower line of the nomogram, which is used to provide individual-level estimates.

Decision curve analyses (DCA) were performed to evaluate the potential clinical utility of the nomogram. DCA evaluates the clinical net benefit at different thresholds by examining the theoretical relation between the threshold probability of an event and the relative value of false-positive and false-negative results^41^.

### Sample size considerations

Given the prevalence of moderate/severe periodontitis of 25%, a random sample of 152 participants including 38 subjects from the positive population and 114 subjects from the negative population produce a two-sided 95% confidence interval with a width of 15% when the sample AUC is 0.875.

## Data Availability

The datasets used and/or analyzed during the current study are available from the corresponding author on request.
